# Decision-Making in the Human-Machine Interface

**DOI:** 10.3389/fpsyg.2021.624111

**Published:** 2021-02-11

**Authors:** J. Benjamin Falandays, Samuel Spevack, Philip Pärnamets, Michael Spivey

**Affiliations:** ^1^Department of Cognitive and Information Sciences, University of California, Merced, Merced, CA, United States; ^2^Scientist at Exponent, Menlo Park, CA, United States; ^3^Department of Psychology, New York University, New York, NY, United States; ^4^Division of Psychology, Department of Clinical Neuroscience, Karolinska Institutet, Solna, Sweden

**Keywords:** mouse tracking, embodied cognition, decision-making, eye tracking, drift diffusion

## Abstract

If our choices make us who we are, then what does that mean when these choices are made in the human-machine interface? Developing a clear understanding of how human decision making is influenced by automated systems in the environment is critical because, as human-machine interfaces and assistive robotics become even more ubiquitous in everyday life, many daily decisions will be an emergent result of the interactions between the human and the machine – not stemming solely from the human. For example, choices can be influenced by the relative locations and motor costs of the response options, as well as by the timing of the response prompts. In drift diffusion model simulations of response-prompt timing manipulations, we find that it is only relatively equibiased choices that will be successfully influenced by this kind of perturbation. However, with drift diffusion model simulations of motor cost manipulations, we find that even relatively biased choices can still show some influence of the perturbation. We report the results of a two-alternative forced-choice experiment with a computer mouse modified to have a subtle velocity bias in a pre-determined direction for each trial, inducing an increased motor cost to move the cursor away from the pre-designated target direction. With queries that have each been normed in advance to be equibiased in people’s preferences, the participant will often begin their mouse movement before their cognitive choice has been finalized, and the directional bias in the mouse velocity exerts a small but significant influence on their final choice. With queries that are not equibiased, a similar influence is observed. By exploring the synergies that are developed between humans and machines and tracking their temporal dynamics, this work aims to provide insight into our evolving decisions.

## Introduction

Human-machine interfaces of various kinds are now ubiquitous in everyday life. For example, purchasing of products frequently takes place via computer, some restaurants use touch screens for ordering from the menu, many voting machines are now electronic, and people spend an inordinate amount of time using their smart phones to interact with their social world (Samaha and Hawi, 2016). In fact, the technology for allowing one’s eye movements to be tracked from a smart phone’s camera has recently been developed (Valliappan et al., 2020). Clearly, a variety of mundane human choices and decisions are no longer being made purely *inside* a human anymore but instead at the *interface* between a human and some form of technology (Clark, 2004). Also, a variety of laboratory human choices and behaviors are now being studied with human-machine interfaces to uncover the mechanics of embodied cognition (Pezzulo et al., 2013; Beckerle et al., 2019). Here, we examine exactly how those choices and decisions can be influenced by that interface.

Decades ago, Gibson (1979) developed the theoretical framework of ecological psychology, which holds at its core the notion that intelligent behavior emerges not from inside an organism but from the interaction between organism and environment (see also, Neisser, 1976; Järvilehto, 1998; Turvey and Shaw, 1999). Thus, the environment surrounding an organism is partly responsible for that organism’s intelligent behavior. If one places that same organism in a different environment, it will produce somewhat different behavior. Around that same time, philosophers of mind were developing the theoretical framework of externalism (Putnam, 1974), which describes mental content as consisting of more than just the information encoded by an organism’s nervous system but also information encoded in the relations that the organism builds with its environment (Clark and Chalmers, 1998; Gallagher, 2017). More recently, cognitive scientists have been developing the theoretical framework of embodied cognition, which includes the brain, the body, and its connections with objects and people in the environment, as the engine of cognitive activity (Barsalou, 1999, 2016; Spivey, 2007; Chemero, 2011). When studying the generation of intelligent behavior, these theoretical traditions encourage one to analyze not just the organism itself but instead the organism-environment system.

When viewed through this theoretical lens, a human reporting a decision via the use of a machine interface is not making that decision inside some encapsulated decision-making module (uninfluenced by context) and then merely recording it (in unaltered fashion) via the machine interface. It is not the case that the organism first makes the decision completely on its own and then reports it via the machine interface. Rather, the dynamic process by which the human interfaces with that machine can influence the decision that eventually gets reached. The decision is not being made by the organism; it is being made by the organism-environment system.

A concrete demonstration of this comes from a study by Pärnamets et al. (2015b), where they presented participants with moral quandaries such as “Is murder ever justifiable?” and gave them response options on a computer screen such as “sometimes justifiable” and “never justifiable.” By recording participants’ eye movements, Pärnamets et al. (2015b) found that participants often fixated one response option and then the other response option and then perhaps back again. Importantly, the amount of time that participants spent looking at their two response options could be used as an indicator of what decision they were about to make before they made it. In Experiment 2, Pärnamets et al. (2015b) further demonstrated that the computer’s timing of its response prompt could slightly influence the choice that the participant made in the end. For each trial, the computer randomly selected a “target” response. Once the participant had fixated that “target” response option for at least 750 ms and had also fixated the non-target response option for at least 250 ms, the computer then urgently prompted a decision from the participant. Thus, at a moment in time when the participant was likely to have been spending more time looking at the “target” response, the computer interrupted the participant’s deliberation and demanded a choice. Without that interruption, it is possible that the participant may have eventually wound up fixating the non-target response option more and finally choosing it. In a decision process that wavers between the two options, “leaning” one way and then the other way, and perhaps back again, this gaze-contingent response-timing manipulation is able to “catch” that decision process at a pre-determined state and trigger a choice based on that state.

In their Experiment 2, Pärnamets et al. (2015b) found that their gaze-contingent response-timing manipulation caused participants to choose the computer’s randomly chosen “target” response option 58% of the time – well above chance. This result suggests that the 2–3 s that a person spends engaged in a wavering decision process while deliberating among two moral choices can be substantively influenced by the manner in which the system they are interfaced with interacts with them. The decision is not being made solely by the human; it is being made by the human-machine interface.

In fact, even with human-human interfaces, this kind of adventitious influence can happen in a way that alters people’s decisions, even moral ones. Consider, for example, a woman who has made a moral commitment to not eating veal anymore, despite the fact that veal parmigiana is her favorite dish. She sits down at her favorite Italian restaurant and peruses the menu. Her eyes flit back and forth between her old favorite, veal parmigiana, and her new replacement, chicken parmigiana. She wants to adhere to her new moral code, but the veal is tempting. Just as her eyes happen to have settled on the veal for about 1 s, suddenly the waiter walks up and asks what she would like to order. If the waiter had shown up a second or two later, she might have managed to settle her eyes, and her mind, back on the chicken. But, in that moment, her decision is prompted and she caves, ordering the veal. Everyday scenarios like this are not very different from the experimental manipulation in the Pärnamets et al. (2015b) experiment.

However, in the Pärnamets et al. (2015b) experiment, a concern can be raised about the 16% of trials which were excluded from the analysis because the gaze-contingent response-timing manipulation was never triggered [see also Tavares et al. (2017) and Newell and Le Pelley, 2018]. On those time-out trials, participants never looked at both response options for the required amount of time to trigger the experimental manipulation. On many of those time-out trials, participants fixated only their internally preferred option and continued to stare at it until the trial was timed-out at 3 s, and then a decision was finally prompted. When those trials were included in the analysis, the overall effect of participants choosing the computer’s randomly chosen “target” response option was reduced (54%) but still statistically significant against a chance level of 50%.

Falandays and Spivey (2020) followed up the Pärnamets et al. (2015b) study with a new set of moral items that were normed for their population to each be very close to equibiased (e.g., near 50/50) in their choices and compared them to non-normed items that were unlikely to be equibiased. This adjustment was meant to address the fact that a subtle influence of gaze on preferences may be washed out by strong, pre-existing preferences for one response option relative to the other (a prediction of the “attentional drift-diffusion model,” discussed in the next section). With no exclusion of time-out trials, the gaze-contingent response-timing manipulation replicated Pärnamets et al. (2015b)’s result, where participants selected the target response 52% of the time – a small but statistically significant effect. With the non-normed stimulus items that were unlikely to be equibiased, no effect was observed.

Ghaffari and Fiedler (2018) replicated and extended the Pärnamets paradigm from moral choices to other-regarding choices. Other-regarding choices are common dilemmas that balance self-interest against the common good. In Ghaffari and Fiedler’s (2018) experiment, they presented participants with pre-recorded spoken queries such as, “If I saw a stranger on the street struggling with her grocery bags, I would help her carry them.” On the computer screen, participants could choose “Only if I have time” or “I would usually help.” In their first experiment, they used a gaze-contingent response-timing manipulation essentially identical to experiment 3 of Pärnamets et al. (2015b), with participants allowed to respond *before* the decision prompt if they so chose. Ghaffari and Fiedler (2018) found that on 38% of trials, participants did, in fact, choose to respond before their eye movements had triggered the gaze-contingent response-timing manipulation. Moreover, 19% of the trials were time-out trials, where the participant’s eye movements did not trigger the gaze-contingent response-timing manipulation over the course of a full 3 s. This left only 43% of trials to have the gaze-contingent response-timing manipulation enacted. Among those trials, the moral-choice trials clearly replicated the findings of Pärnamets et al. (2015b), but the other-regarding trials did not. However, the same concern remains, as before, regarding the exclusion of trials in which the gaze-contingent response-timing manipulation was not triggered. Analyzing only that subset of trials introduces a biased selection problem (Newell and Le Pelley, 2018).

Therefore, in Ghaffari and Fiedler (2018) second experiment, instead of a gaze-contingent response-timing manipulation, the response options turned on and off on the screen in a manner that simulated the fixation patterns of previous participants. The “target” response option, in this case, was the option that had been chosen by that previous participant. Importantly, no exclusion of trials was needed in this paradigm. Again, corroborating the pattern observed by Pärnamets et al. (2015b) and Ghaffari and Fiedler (2018) found that, in the moral-choice trials, participants chose the target option more often (53% of the time). However, with other-regarding trials, no effect was observed.

With moral-choice items, at least, Ghaffari and Fiedler (2018) concluded that while the majority of the variance in a decision-making process may rest in the hands of top-down cognitive processes, some portion of that variance is controlled by events that take place in the perception-action cycle of a person interacting with their environment. Their preferred model for this combination of top-down and bottom-up influences is the attention-drift-diffusion model (aDDM) developed by Krajbich et al. (2010; see also Krajbich, 2019).

## The aDDM: How Visual Attention Influences Decision-Making

The drift diffusion model (DDM) is a standard model for simulating choices and response times in a two-alternative forced choice task, which assumes that decisions are made through the stochastic accumulation of perceptual evidence until a decision threshold is exceeded (Ratcliff and McKoon, 2008). The standard DDM represents the relative evidence for one of two alternatives at time *t* as *x(t)* according to the following equation (Bogacz et al., 2006):

(1) $$x_{t+1}=x_{t}+A+W$$

When *x* is 0, the two options have equal relative evidence, whereas positive or negative values indicate greater evidence for one option than the other. The change in evidence over time is the result of a constant “perceptual evidence” factor, *A*, plus Gaussian noise, *W*. For an initially undecided choice, *x* = 0 at *t* = 0, indicating equal support for each of the two response options.

An arbitrary upper and lower bound are set, such that when *x* crosses either boundary, a decision is made in favor of the corresponding response option. For example, if the reference option is coded as a +1 and the alternative option is coded as −1, if *x* crosses the boundary at −1 the model is treated as having chosen the alternative option. Due to the presence of noise, DDMs offer an account for why participants will sometimes choose response options with lower relative value. When the magnitude of *A* is small (one option is only slightly preferable to the other) and/or *W* is large, drift due to noise can dominate the decision.

While the standard DDM is designed to represent perceptual decisions based on a single stimulus, Krajbich et al. (2010; see also Pärnamets et al., 2014) adapted this model to the context of choosing between two displayed stimuli through visual sampling. Their attention-drift-diffusion-model (aDDM), which provided a close fit to human data, allowed the rate of evidence accumulation to vary as a function of the currently fixated option. This represents a cognitive discounting of the value of currently un-fixated options. The simple intuition here is that a response option that is “out of sight” is also (at least partially) “out of mind.” For this version of the model,

(2) $$x_{t+1}=\left\{ \begin{array}{c} x_{t}+d\left(A_{left}-\theta A_{right}\right)+W,leftisfixated\\ x_{t}+d\left(\theta A_{left}-A_{right}\right)+W,rightisfixated \end{array}\right.$$

where θ is a value between 0 and 1, which discounts the value of the currently unfixated option, *d* represents the rate of information accumulation, and *W* represents Gaussian noise.

Given the reasonable assumption that visual attention biases information accumulation, and that decision outcomes depend upon accumulated information, it follows that one can influence decision outcomes by influencing the gaze. Because an advantage is conferred upon fixated response options, options that are fixated for longer should be more likely to be chosen. Importantly, however, this effect is dependent upon both the magnitude of cognitive discounting on non-fixated options, as well as the relative values of the two response options. Examining Equation 2, we can see that if the value of *A*_unfixated _is large relative to *A*_fixated _, discounting would need to be substantial (θ*near* 0) in order for gaze to change the direction in which preferences evolve. Under normal circumstances, we can assume that participants at least momentarily fixate, and therefore have some awareness of, both response options, so we would expect discounting to be less than complete. As such, the aDDM only predicts a meaningful role of gaze when the two response options are roughly equal in value (see also Tavares et al., 2017).

To demonstrate how a human-machine interface may influence decisions by exploiting the dynamics of visual attention, in this section we present a simplified simulation of the experimental task in Pärnamets et al. (2015b), using an adapted version of the aDDM. To provide a point of comparison, we also conducted a replication of Pärnamets et al. (2015b) with human participants, with the only change being that our moral stimuli were normed to be equibiased in our population (obtaining 50 ± 10% agreement), while our non-moral stimuli were taken directly from the set used in Pärnamets et al. (2015b) and were not normed for our population, and therefore were unlikely to be equibiased^1^.

### Methods

Our adapted version of the aDDM begins with the equation introduced by Krajbich et al. (2010; Equation 2). In modeling gaze behavior, we adopted the following simplifying assumptions: (1) one alternative is fixated at any given time, (2) the first fixation on any trial is random, (3) there is a minimum fixation length, after which fixation switches are determined by competition between current preferences and attentional fatigue, and (4) saccades are instantaneous. The minimum fixation time was set at 200 timesteps, representing 200 ms, which is approximately the time required to plan and launch a saccade (Salthouse and Ellis, 1980). After this period, attentional fatigue *f* begins to accumulate at a constant rate *d_f_:*

(3) $$f_{t+1}=\left\{ \begin{array}{c} 0,consecutivefixations<200timesteps\\ f_{t}+d_{f},consecutivefixations>=200timesteps \end{array}\right.$$

Attention, *a*, was modeled as deviating from the value of current preferences, toward the alternative option, by the current magnitude of *f* until the 0-line is crossed, inducing a switch in gaze:

(4) $$a_{t}=\left\{ \begin{array}{c} x_{t}-f_{t},x_{t}>0\\ x_{t}+f_{t},x_{t}<0 \end{array}\right.$$

**FIGURE 1:**
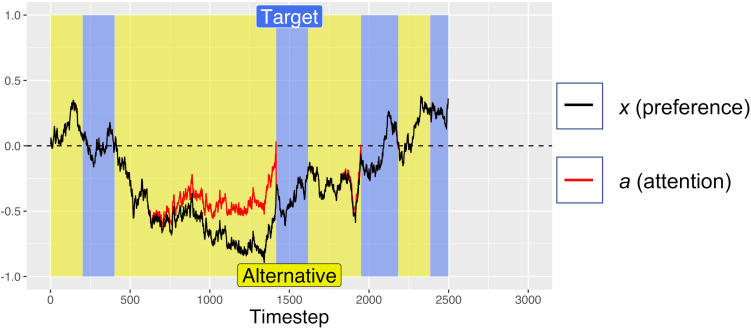
An example simulate dtrial with no initial preference (*A*_target_ = *A*_alternative_ = 0.5) in which the model was trending toward preferring the non-target alternative, but once it satisfied the gaze-contingent response-timing criteria, it selected the target. Periods of fixating the target are marked in blue, with fixations to the alternative in yellow. After a minimum fixation time of 200 timesteps, attention begins to decay toward the currently unfixated option (clearly visible between ∼500 and 1400 timesteps), triggering a switch in gaze when crossing the zero-line. Also note how accumulation of preference is biased toward currently fixated options (e.g., the strong trend toward the alternative option from ∼500 to 1400 ms, while fixating the alternative). For this simulation: *d* = 0.001, *d*_f_ = *0.0005*, and *W* was Gaussian noise with mean 0 and s.d. of 0.01.

**FIGURE 2:**
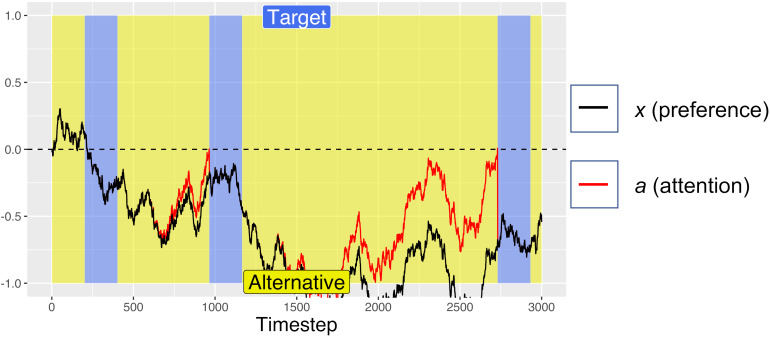
An example simulated trial with no initial preference (*A*_target_ = *A*_alternative_ = 0.5) on which the simulation “timed-out” after 3 s (because cumulative target fixation time <750 timesteps), then selected the non-target alternative. Periods of fixating the target are marked in blue, with fixations to the alternative in yellow. For this simulation: *d* = 0.001, *d*_f_ = *0.0005*, and *W* was Gaussian noise with mean 0 and s.d. of 0.01.

For the first 200 timesteps of any fixation, *a* is exactly equal to *x*, but after this time, attentional fatigue causes *a* to move toward zero. When *a* crosses the zero-line and changes sign, this represents an attentional switch, and gaze is directed toward the previously unfixated option. Because *a* is coupled to *x* (attention is coupled to current preferences), greater magnitudes of *x* can offset the decay from *f*, such that the model looks longer at options that it “prefers,” despite some attentional fatigue. Similar attentional parameters are commonly used in dynamical systems models of bi-stable perceptual phenomena, such as the Necker cube, to account for perceptual reversals (Ditzinger and Haken, 1995; Fürstenau, 2014). The red lines in Figures 1, 2 show how attention decays as compared to decision preference (black lines), leading to gaze-changes (alterations between blue and yellow regions). Note that, while attentional fatigue can lead to gaze switches, unless there is a corresponding switch of preferences, gaze will switch back to the preferred option after the minimum fixation time (e.g., in Figure 1, the second blue section, a brief period of fixating the target from ∼1400 to 1600 ms).

On each simulated trial, the pre-designated target was randomly assigned to one of the two response options. Each trial was run for a maximum of 3000 timesteps, analogous to the 3 s time limit in the Pärnamets paradigm. We recorded the number of timesteps spent “fixating” each alternative. If at least 750 timesteps of gaze accumulated on the target side and at least 250 timesteps on the alternative side (analogous to the 750 ms/250 ms threshold in the experiment), the trial was ended, and a positive *x* value resulted in choosing the reference option (coded as +1) while a negative *x* value resulted in choosing the other option (coded as −1). Figure 1 shows an example trial where the simulation met the gaze-time thresholds after 2500 timesteps and selected the target option (because *x* was >0 at the end of the trial). Figure 2 shows an example trial where the simulation did not fixate the target for long enough to satisfy the gaze-contingent response-timing criteria, leading to a time-out after 3000 ms, after which the simulation selected the non-target alternative.

### Results

This simple model was not intended to precisely characterize psychometric variables in our population, but rather to show that drift diffusion models straightforwardly predict the pattern of results obtained when using biased or equibiased stimuli. Thus, to avoid overfitting, no parameter tuning was done. The gaze-bias parameter (θ) was set to 0.5, such that the currently unfixated option was discounted by half. The rate of information accumulation (*d*_A_) was set to 0.001 and the gaussian noise (*W)* was set to a mean of 0 and a standard deviation of 0.01. The rate of attentional fatigue accumulation (*d*_f_) was set to 0.0005. To simulate our normed equibiased stimuli, we set the values of both options to 0.5. To simulate biased stimuli, we set the values of one option to 0.8 and the other to 0.2, randomly assigned on each trial. 50000 simulated trials were run for each set of values. For each trial, we recorded the choice made by the model (determined by the sign of *x* when the trial terminated) as well as whether or not the trial “timed-out” by reaching 3000 ms without meeting the gaze-time criteria.

The general behavior of this simulation approximates the data in Falandays and Spivey (2020) remarkably well, especially given that we have not systematically explored the parameter space with this model. The results summarized in Table 1 show that the differences across equibiased or biased stimuli in the simulation are in the same direction and have similar magnitudes to the trends across moral and non-moral stimuli in the human experiment, despite some differences in absolute values.

**TABLE 1:** Summary results from the aDDM simulation and human experiment.

	Simulation results		Human results	
	Equibiased	Biased	Moral	Non-moral
Prop. timeout trials	40.57%	75.84%	23.59%	64.87%
Prop. target choices, all trials	56.13%	52.03%	52.3%	49%
Prop. target choices, non-timeout trials	79.69%	89.02%	62.67%	65.67%
Prop. target choices, timeout trials	21.63%	40.25%	20.18%	40.16%

### Discussion

The results of this simple drift diffusion model provide a close approximate match to the results obtained from human participants. The relatively uncertain moral stimuli, exhibiting minimal intrinsic cognitive bias toward either of the response options, produced time-out trials less than half of the time, whereas the intrinsically biased filler stimuli produced time-out trials more than half of the time. When these time-out trials were excluded from analysis, both moral and filler stimuli exhibited strong choice preferences for the pre-designated target response, just as seen in our human data. However, when time-out trials were included in the analysis, only the moral items showed a reliable preference for the pre-designated target response – again, just as seen in the human data. Thus, results from the human data (Falandays and Spivey, 2020), and from this aDDM simulation, demonstrate that the gaze-contingent response-timing manipulation is most effective with queries that have relatively equibiased options, and less effective with queries that have substantial pre-existing biases.

A novel theoretical contribution of this model concerns the role of attentional fatigue. Given that attentional fatigue can result in switches of gaze, and gaze discounts non-fixated options, it follows that attentional fatigue can slow the accumulation of evidence toward a higher-valued option. For example, if a participant prefers option A, and therefore fixates option A, attentional fatigue may eventually divert their gaze to option B. Given a minimum fixation time before launching a saccade, option A will be temporarily cognitively discounted. If the options are relatively equibiased, temporary gaze switches due to attentional fatigue may be enough to tip the scales of evidence/preference toward option B.

## The mDDM: How Motor Costs Can Influence Decision-Making

Importantly, from the theoretical position we are advancing here, visual attention is by no means a privileged influence upon decision-making. In this section, we address the influence of an entirely different factor in the decision-making process: the relative costs, in terms of time and/or effort, associated with making different response options. Consider the example of perusing a shelf at the grocery store, looking for ingredients for a recipe. You may notice that a cheaper, generic brand of ingredient is positioned on the top-most shelf, and would be a bit difficult to reach. If you’re in a rush, you instead might choose to grab the slightly more-expensive version directly in front of you, even though you know the quality is no better. Marketers are of course well aware of phenomena such as this, and compete to put their products in the most visible, convenient locations (Gidlöf et al., 2017). There is no reason to think that similar biases couldn’t be implemented in human-machine interfaces, including the increasingly ubiquitous case of making decisions with a computer mouse.

A key insight of work using “process-tracing” techniques to study decision-making – techniques which take many samples of a behavioral variable over a short period of time, such as motor movements or gaze position – is that mental representations, such as attitudes and preferences, do not spring to the mind as fully formed, discrete entities (Spivey, 2007). Instead, explicit reports of attitudes are merely the discrete output at the end of a continuous cognitive process, one that dynamically evolves on the scale of milliseconds and seconds in decision-making (Wojnowicz et al., 2009), or over longer periods in development (Smith and Thelen, 2003). For example, Wojnowicz et al. (2009) presented participants with various nouns and had them indicate whether they “like” or “dislike” the presented stimulus, using their mouse-cursor to select their response. In some cases these nouns were things that most people uncontroversially like, such as “sunshine,” or dislike, such as “murderers.” But the key stimuli probed participants’ implicit biases: they were “white people” and “black people.” The authors proposed that, if participants had implicit biases against the latter group, the unfolding movements toward the chosen response option would show evidence of some cognitive dissonance, even when the end result of the decision process was the same for both stimuli (reporting “like” for both groups). Indeed, the authors found that the trajectories of the mouse-cursors made relatively straight paths toward “like” when the stimulus was “white people,” but curved more toward “dislike,” before eventually landing on “like,” when the stimulus was “black people.” Beyond the disheartening evidence of implicit racial biases, this result also shows that multiple, conflicting attitudes may be simultaneously “competing” for control of the decision-making process as it unfolds over time.

Response deadline procedures (Dosher, 1976) also provide some insight into these partially active representations that are simultaneously active at early moments of the unfolding decision-making process. For example, McElree et al. (2006) induced speeded True-False responses to sentences such as “Water pistols are harmless,” using response deadlines of 300, 500, 700, 900, 1500, and 3000 ms. They found that the short deadlines elicited a substantial number of incorrect responses with d-prime increasing non-linearly over time. Similarly, Spivey et al. (2002) induced speeded sentence completions for fragments such as “The patient cured…” and “The judge sentenced…,” using response deadlines of 300, 600, 900, and 1200 ms. They found that the short deadlines elicited a greater proportion of the rare relative clause completion, e.g., “The patient cured by the doctor was happy.” By contrast, with those sentences, the longer deadlines elicited almost exclusively the common main clause completion, e.g., “The patient cured himself” (Spivey, 2007, chapter 7). For decades, results like these have suggested that multiple competing representations are simultaneously partially active early on during a cognitive process and this activation pattern evolves into singular decision over the course of several hundred milliseconds.

The dynamic evolution of decisions on a short time scale is represented in the DDM as the accumulation of evidence or preference. This process can of course be *willfully* biased by the decision-maker, for example by adopting a liberal or conservative response criterion under differing task demands. For example, Kloosterman et al. (2019) found that liberal response criteria are associated with a suppression of alpha band activity, relative to conservative criteria, which appears to systematically bias the direction of evidence accumulation toward a “target present” response in a go/no-go task (see also: Kloosterman et al., 2020). But given the logic of the DDM, influencing neural activity through explicit task demands is only one method of introducing systematic bias into the decision-making process, and other mechanisms may be external to the cognitive dynamics of evidence accumulation entirely. For example, the experimental manipulation of Pärnamets et al. (2015b) works by using gaze to probe the likely balance of evidence over time in the evidence accumulation process, and requiring a decision when evidence is expected to favor a pre-chosen “target” option. Another mechanism would be to introduce asymmetrical costs in making different response options, which may consciously or unconsciously factor into response criteria. The grocery store example mentioned above is one flavor of this: individuals may systematically discount the value of options that are more difficult to choose.

There is already some evidence that *perceptual* decisions can be influenced by the motor cost of responding. Hagura et al. (2017) instructed participants to move either a left or a right lever to indicate the direction of coherent motion using standard random-dot motion stimuli. During training, the researchers gradually increased the resistance on one lever relative to the other, such that one response required more force and thus became more costly. The authors found that participants then required *greater* perceptual evidence before making the more difficult response. Interestingly, based on a comparison of model fits, the authors concluded that the motoric cost of action directly influenced the perceptual stage, rather than influencing the participants’ response criterions.

The study by Hagura et al. (2017) can be seen as an extension to the domain of perceptual decision-making of prior work on motor control by Shadmehr et al. (1993) and Shadmehr and Mussa-Ivaldi (1994). In this earlier work, participants held a robotic manipulandum that either displaced the hand from an equilibrium starting position (Shadmehr et al., 1993) or altered the forces acting on the hand through some regions of space during reaching movements (Shadmehr and Mussa-Ivaldi, 1994). The forces acting to displace the hand constitue a motoric “force field.” Shadmehr and colleagues found that participants adapted to the displacing force fields of the robotic manipulandum through restorative movements, which defined a *postural* force field complementary to the one applied by the manipulandum. Furthermore, Shadmehr and Mussa-Ivaldi (1994) found that with a substantial amount of training, participants were able to adapt to the presence of a displacing force field during reaching movements, achieving movement trajectories similar to those seen in the absence of a force field prior to training. However, after some training with the force fields, when these fields were suddenly removed, participants “over-corrected” in their movements, applying restorative forces when none were needed. Based on these results, (Shadmehr and Mussa-Ivaldi, 1994) concluded that participants accomplish this reaching task by adjusting an internal model of the movement dynamics of the hand, arm, and shoulder, which predicts the forces that will be encountered over the course of a movement.

But motor costs need not be explicitly calculated nor implicitly learned in order to influence the outcome of decisions. Given the logic of the DDM, it follows that merely increasing the time it takes to reach one outcome relative to another can also bias outcomes (under the additional assumption that individuals continue to accumulate evidence, rather than endogenously stopping the process). Because noise plays a role in the accumulation of preference over time, decision outcomes that take longer to achieve (therefore drawing out the evidence accumulation process) allow more time for noise to tip the scales toward alternative outcomes. To show how this may be the case, in this section we present another variant of the DDM, which we will call the motor-drift-diffusion-model (mDDM). Using this model, we consider the case of an individual making a decision using a mouse cursor, but where a subtle bias in the cursor operation makes it easier to move in one direction than another.

### Methods

The present model made one small change to the standard DDM (Eq. 1) with the inclusion of a second variable, *m*, representing the current position of a mouse cursor (or it could represent a hand, or an entire body) moving along a single dimension toward one of two response options. The model was treated as having made a decision only when *m*, rather than *x* (the evidence or preference variable), reached the upper or lower bound of ±1, which represents a participant moving the mouse to the left or right top corner of a screen, and concluding a trial by clicking one response option. Meanwhile, *x* was bounded between the values ±1 and, unlike in the aDDM, *x* reaching either boundary had no effect on terminating the trial. This means that the model could attain maximum preference for one response option, yet the preference accumulation process would not terminate until an *action* threshold was also met.

**FIGURE 3:**
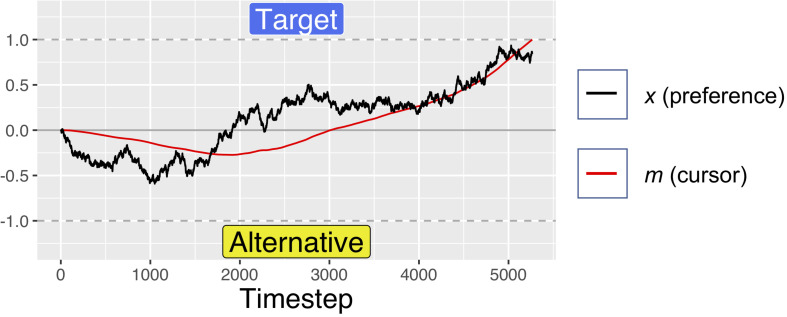
A simulated trial with no initial preference for either response option. The evolution of the decision value is driven only by noise, but the velocity squashing effect makes movements toward the alternative slightly slower. This makes it more likely for noise to result in movements drifting toward the target. For these simulations, *d* = 0.001, *s* = 0.45, and *W* was Gaussian noise with mean 0 and s.d. of 0.01.

**FIGURE 4:**
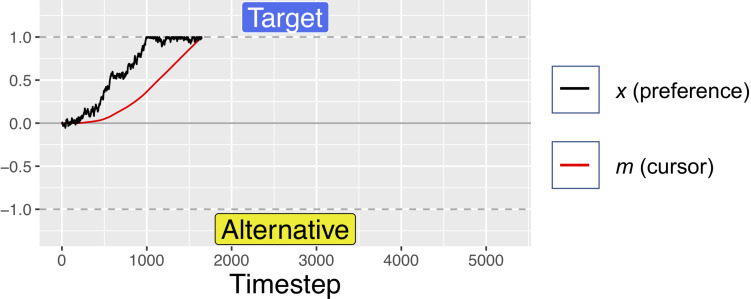
A simulated trial where the target is fully preferable to the alternative. Notice how the black trajectory (representing evolving preference) in this figure and Figure 5 below reach the boundary at approximately the same time, but the red line (representing the cursor) reaches the boundary faster here than below. For these simulations, *d* = 0.001, *s* = 0.45, and *W* was Gaussian noise with mean 0 and s.d. of 0.01. For comparison of response times, this figure uses the same *x*-axis as Figure 3, but note that this trial ended at ∼1500 timesteps.

**FIGURE 5:**
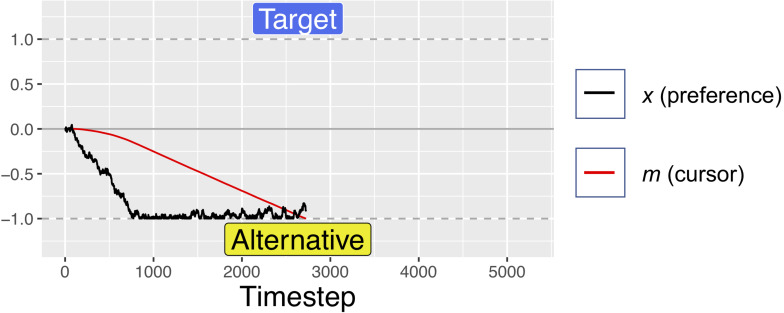
A simulated trial where the alternative is fully preferable to the target. Notice how the black trajectory in this figure and Figure 4 above reach the boundary at approximately the same time. However, here the red line lags further behind the black trajectory as a result of the velocity squashing effect, because movements are toward the alternative. For these simulations, *d* = 0.001, *s* = 0.45, and *W* was Gaussian noise with mean 0 and s.d. of 0.01. For comparison of response times, this figure uses the same *x*-axis as Figure 3, but note that this trial ended at ∼2750 timesteps.

The position variable *m* was computed by integrating the preference variable *x* at rate *d.* This results in the *m* moving toward the currently preferred response option with a velocity determined by the magnitude of current preferences. As can be seen in Figures 3–5, this simple mechanism produces smoothly changing position curves from the noisy preference signal, which are reminiscent of the movement trajectories seen in mouse-tracking studies with similar designs (e.g., Spivey et al., 2005). As in our previous model, on each simulated trial, one of the two response options was designated as the “target” option – the option that the software “wants” the simulated participant to choose. For simplicity, the target was always designated as +1, and the alternative was designated as −1. Meanwhile, the relative values of the target and alternative options were allowed to vary from trial to trial.

Critically, the *m* variable was also influenced by a directionally dependent velocity bias. When *x* was on the side associated with the target (i.e., the model currently prefers the target and therefore is moving toward it), the change in *m* was equal to *x*. On the other hand, when *x* was closer to the non-target alternative, the change in *m* was equal to x multiplied by *s*, a velocity squashing factor. Formally stated:

(5) $$m_{t+1}=\left\{ \begin{array}{c} m_{t}+dx_{t},x_{t}>0\\ m_{t}+sdx_{t},x_{t}<0 \end{array}\right.$$

Based on these functions, the magnitude of current preferences determines the velocity toward the preferred option (see also, Abrams and Balota, 1991). However, the velocity is squashed by some proportion for movements in the direction of the computer’s pre-designated non-target alternative, making movements toward the non-target slower (see Figure 7; also see Figure 8 for a schematic illustration of this mechanism for the human experiment). Because the relative decision value variable *x* is bounded between −1 and +1, velocity is bounded at *d* units per time step.

**FIGURE 6:**
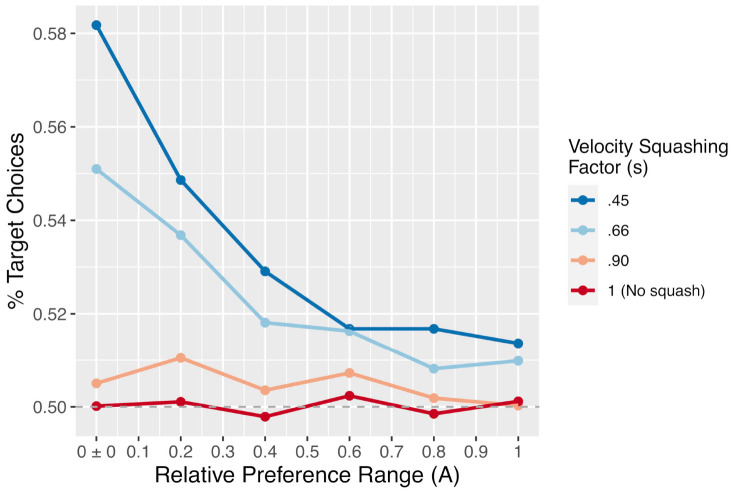
The proportion of trials on which the simulation chose the target option as a function of the velocity squashing factor and the width of the distribution from which preferences, *A*, were drawn. Each data point is the result of 50,000 simulated trials.

**FIGURE 7:**
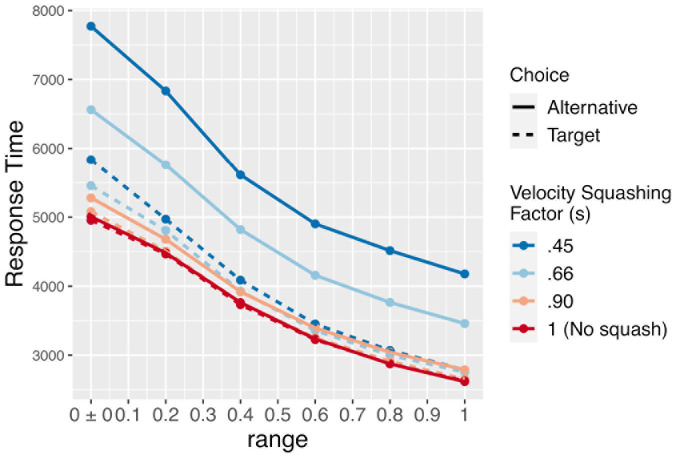
Mean response times for the simulation (in timesteps, roughly equivalent to ms) by the size of the velocity-squashing manipulation and whether thee model chose the target or alternative option. Response times are faster when choosing the target due to the velocity squashing manipulation slowing movements toward the alternative.

**FIGURE 8:**
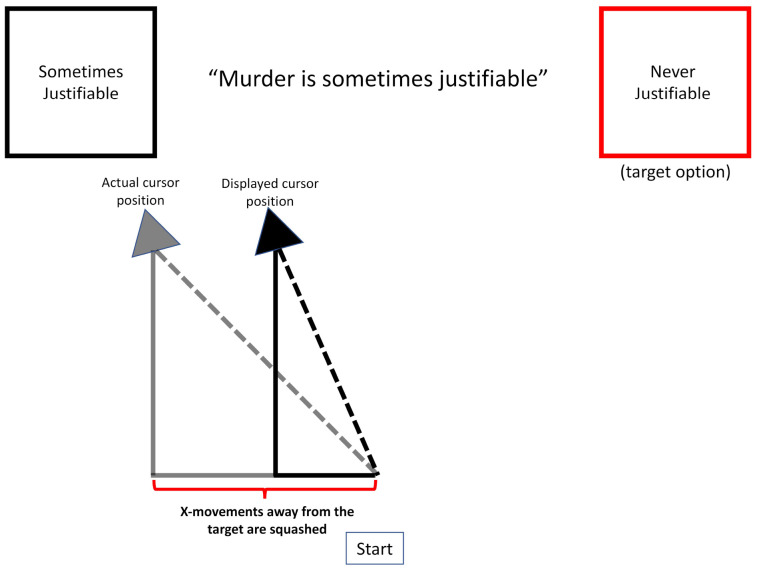
A schematic of the operation of the velocity-squashing manipulation. The change in x-position for movements *away* from the target are squashed by a factor of 0.45 and the cursor is reset to a new position. For example, on this trial, a cursor movement of 10 pixels to the left would result in a displayed movement of 4.5 pixels to the left. Movements *toward* the targets are unperturbed.

We explored three different sizes of the velocity squashing effect, s, in the simulation:0.45, 0.66, and 0.90. We also simulated the case of no effect (or a velocity squashing factor of 1) as a control. While varying the velocity squashing parameter can be informative as to the general patterns that occur as the effect varies in strength, it is important to note that real time and space do not map clearly onto simulated time and space in these models, nor are we using a highly realistic model of movement generation. As such, it is not necessarily the case that a squashing factor of 0.45 will produce the exact same effects in the simulation as in the human experiment that follows in the next section.

To explore how the effect size varies as a function of how balanced of a stimulus set is used (meaning whether the two response options in each pair tend to be near equally preferable), we varied the distribution from which the *A* parameter was drawn. Recall that the *A* parameter determines the preferability of the target reference option relative to the non-target alternative. We defined this as a value between −1 and +1, where positive relative preference value indicate preference for the response option coded as +1, while negative values indicate preference for the option coded as −1. On each simulated trial, the value of *A* was drawn from a uniform distribution bounded within some range. We explored ranges from ±0 to ±1 in increments of 0.2; such as 50/50, 60/40, and up to 100/0. Examples of simulated trials with no difference in preferability of the options, maximum preference for the target, and maximum preference for the alternative are shown in Figures 3–5. For readers interested to get a greater sense of how the velocity squashing parameter influences movement dynamics in each of these cases, the Supplementary Material contains GIF files plotting dynamic time series for 10 random trials with each of the parameter settings used for Figures 3–5.

At the start of each simulated trial, either the +1-coded or −1-coded response option was randomly designated as the target option. Thus, when the target was the +1 option, movements in the negative direction were squashed by *s*, and vice versa when the −1 option was the target. Each trial proceeded until *m* crossed the upper or lower limits of ±1, and the model was treated as having chosen the response option with the corresponding code. For example, when the model crossed the upper boundary, it was treated as having chosen the +1 coded option. These choices were then compared against the target option on that trial and recoded as a target or non-target choice. In keeping with our previous implementation of the aDDM presented earlier, the rate of information accumulation *d* was set to 0.001, and gaussian noise *W* was introduced at each time step, with a mean of 0 and standard deviation of 0.01. The model was run for 50,000 simulated trials for each combination of stimulus parameters, and each trial was allowed to proceed until *m* crossed the upper or lower boundary.

### Results

The primary results of the simulation experiment are shown in Figure 8. As the figure shows, there is a clear main effect of the velocity squashing factor, with a greater likelihood of choosing the pre-designated target option seen when movements away from the target are reduced by a greater proportion (i.e., as they are multiplied by a smaller squashing factor). Importantly, the choices are at chance (50%) when the velocity squashing manipulation is turned off (*s* = 1, the red line in Figure 6). There is also an effect of the width of the distribution from which the relative preferences were drawn – the *A* parameter in our model – whereby the effect size of the squashing manipulation diminishes as the range of *A* values increases, meaning as stimuli become more biased with respect to initial preferences. Finally, there is an interaction between these two parameters such that the effect of *s* diminishes more quickly for smaller values of *s* (stronger manipulations).

Importantly, motor costs are not calculated and factored into decisions in this model. Instead, the effect of the manipulation is attributable to a bias in the accumulation of noise in preference, as it is mapped onto movements. When noise induces a change in preferences toward the target option, movements in that direction are faster, which increases the likelihood of reaching the target and terminating the trial. This is evident in the faster response times of the simulation when selecting the target vs. the alternative (solid vs. dashed lines in Figure 7). The response times also show a clear effect of the bias in initial preferences (the width of the distribution from which *A* is drawn). When *A* is small and stimuli are relatively equibiased, the signal-noise ratio becomes weaker, and when *A* = 0, the evolution of preferences is driven entirely by noise. This increases the mean time required to reach a response, and thereby increases the opportunity for noise to accrue in movements toward the target.

### Discussion

This simple model demonstrates how, by slowing down movements away from the target option, our manipulation biases the effect of drift due to noise in the direction of the target, resulting in more trials on which the simulation ultimately chooses the target.

As Figure 6 illustrates, while the effect diminishes in size as pairs of response options become less equibiased (i.e., increasing the range of *A*), the effect does not disappear completely even for a subtle velocity squashing effect and relatively biased stimuli. This result can be contrasted to the findings of Falandays and Spivey (2020) regarding the gaze-based timing manipulation, which showed that the effect may disappear completely when the response options are not equibiased. The difference between these two manipulations may be explained by the fact that the gaze-based response-timing manipulation, used by Pärnamets et al. (2015b) and others, is imposed only when participants spend time fixating both response options, whereas the present motoric manipulation is active on all trials. As such, this velocity bias may be able to subtly influence decisions even when one response option is strongly preferred relative to the other. Note, however, that the strongest effect observed in the simulation, a ∼58% preference for the pre-designated target, is obtained only with a perfectly equibiased stimulus and an extreme velocity squashing. In a human experiment with stimuli that are *approximately* equibiased (but not perfectly so) and velocity squashing that is mild enough to go undetected, the effect may be substantially smaller than that.

## Experiment: Manipulating Decisions With Mouse Cursor Velocity

We next sought to test the predictions of our mDDM in a human-subjects experiment. In this experiment, we asked whether a subtle bias in the movement of a mouse cursor could push decisions toward a randomly pre-selected option. The present experiment was a conceptual replication of Pärnamets et al. (2015b), and used the same stimuli as a recent replication of that experiment, Falandays and Spivey (2020). On each trial, participants first heard a moral or non-moral (factual, opinion) statement or question, then two possible response options appeared in boxes in the top-left and top-right corners of the screen, respectively. In those previous experiments, participants responded after a prompt screen appeared, using one of two key presses to respond. However, in the current study, participants responded with the mouse cursor by moving it from a central starting point near the bottom of the computer screen to click on their chosen response option at the upper corners of the computer screen. The response options remained on screen until one was selected. As before, on each trial, one response option is randomly pre-designated as the “target” – the option we are trying to bias their decision toward. Unbeknownst to the participant, the experiment software acted to subtly decrease the velocity of the mouse cursor for any movements *away* from the target option (or toward the non-target), such that the mouse moved slightly slower toward one option than toward the other. Based on the mDDM simulation above, we predicted that this motoric manipulation would result in participants choosing the target option slightly more often than chance and would influence even stimuli for which the response options were not equibiased.

As we briefly discussed earlier, our velocity squashing manipulation is similar to the “force fields” used by Shadmehr et al. (1993) and Shadmehr and Mussa-Ivaldi, 1994 to investigate human motor control. In light of the adaptation to force fields observed by Shadmehr and Mussa-Ivaldi (1994), it might be predicted that human participants could also adapt to our motor perturbations, resulting in no effect of the manipulation on choices. However, a key difference between our paradigm and that of Shadmehr et al. (1993) and Shadmehr and Mussa-Ivaldi (1994) as well as that of Hagura et al. (2017) is that the direction of the force field was randomized across trials, which would likely preclude a generalized adjustment of an internal model of reaching dynamics. For this reason, our model did not include any calculation of motor costs or adaptation on that basis. Instead, we propose that force-field-like manipulations can also bias decision-making in the absence of adaptation, simply by increasing the time it takes to reach one response option relative to the other, and thereby allowing greater opportunity for noise to push decisions toward the target option.

### Method

#### Participants

Eighty healthy undergraduate students (61 female, 18 male; age: mean ± s.d. = 19.3 ± 1.37) were recruited from the subject pool at the University of California, Merced. Participants provided informed consent in accordance with IRB protocols and received course credit for their participation. Participation was restricted to those who reported being right-handed, having normal or corrected-to-normal vision and hearing, and not having a reading disorder or physical disability that would prevent simple actions with the hands.

#### Materials

The stimuli consisted of 72 prompts with two response options per prompt. The stimulus selection procedure is reported in Falandays and Spivey (2020), and the full set of stimuli is available on our preregistration page on the Open Science Framework^2^. Half of the prompts consisted of a statement expressing an opinion on some moral or ethical issue (e.g., “Murder is sometimes justifiable.” Or “Hunting for sport is OK if it doesn’t harm the ecosystem”). These stimuli were normed to generate 50 ± 10% agreement in our population. Because these stimuli were designed with the explicit goal of generating uncertainty and conflict in choosing, response options did not necessarily represent the extreme endpoints of an opinion spectrum. For example, in response to the statement “Murder is sometimes justifiable,” the extreme opinion endpoints might be “Never justifiable” and “Always justifiable,” but in this case the latter response is expected to be universally undesirable, and therefore these two options would be unlikely to generate uncertainty and conflict.

The other half of the prompts were non-moral filler questions regarding opinions or facts (e.g., “Do people respect selflessness?” or “Can bacteria live in boiling water?”) on which no norming was conducted. Response options to these stimuli were always “Yes” or “No.” As they were in the studies of Pärnamets et al. (2015b) and Newell and Le Pelley (2018), these non-moral items are considered “filler” items and are included mainly to prevent participants from focusing exclusively into a mindset of moral reasoning. In principle, the filler items may also show an effect of the gaze-based timing manipulation. However, given that these stimuli were not normed to be near 50/50 uncertainty, given that the word length of the response options is much shorter than those in the moral condition, and given that the response options are identical for all filler items, we expected these items to be relatively far from equibiased (represented in the DDM as *A* > > 0). In Falandays and Spivey (2020), these items showed no effect of the gaze-contingent response-timing manipulation (once time-out trials were included). However, based on our mDDM simulations above, these items may nonetheless be susceptible to this computer-mouse velocity manipulation and may reveal a subtle preference for the pre-designated target response option.

Stimulus queries were presented auditorily over headphones at the participants’ preferred volume. Response options consisted of white text centered in a 300 × 300 pixel white box on a black background. Boxes were placed in the top left and top right corners of a 1920 × 1200 pixel screen, with a 30 pixel buffer between each box and the closest edge of the screen. Text was displayed in Times New Roman size 70 font.

#### Procedure

Participants completed the experiment individually in the lab, in a single session taking approximately 30 min. Participants were seated in front of a computer and wearing headphones with the volume set to their most comfortable level. The experiment was run using the Psychophysics Toolbox package (Brainard, 1997) in Matlab. On each trial, a white fixation dot was displayed in the center of the screen while the audio prompt played over the headphones. During this time, the mouse cursor was made invisible. Once the auditory prompt finished playing, the two response options would appear in the top-left and top-right corners of the screen. Upon completion of the query and appearance of the response options, the mouse cursor was reset to the bottom center of the screen, and participants moved the mouse to click on their choice. Participants had no time limit to make a selection. The left or right position of each response option was randomized. On 36 randomly selected trials, the left option was selected as the target, while the right option was selected on the remaining 36. After each trial, participants rated their confidence in their choice as well as their understanding (the degree to which they read and understood both response options) on a 1–7 scale.

In order to manipulate the motor cost of responding in an asymmetric fashion, the computer program made it slightly more difficult for participants to move the mouse cursor away from the target than toward it. This was accomplished by doing a fast re-draw of the mouse cursor position. Every 10 ms, the change in the x-position was sampled. Changes in the direction away from the target were squashed by a factor of 0.45, such that the mouse cursor was repositioned closer to its origin (along the *x*-axis) than it had actually traveled. This resulted in a decreased velocity when moving in one direction, though which direction was impacted was random across trials.

After completion of the main phase of the experiment, participants were presented with two free-response questions designed to probe for detection of the experimental manipulation in the experiment. The first question asked “What do you think was being manipulated in this experiment?” The second question asked “Did you notice anything unusual about the functioning of the computer program used in this experiment?” An experimenter was present to offer participants clarification on the meanings of the questions, when necessary.

#### Data and Code Availability

All code and data from this experiment and preceding simulations are available are available on the Open Science Framework^3^.

### Results

**FIGURE 9:**
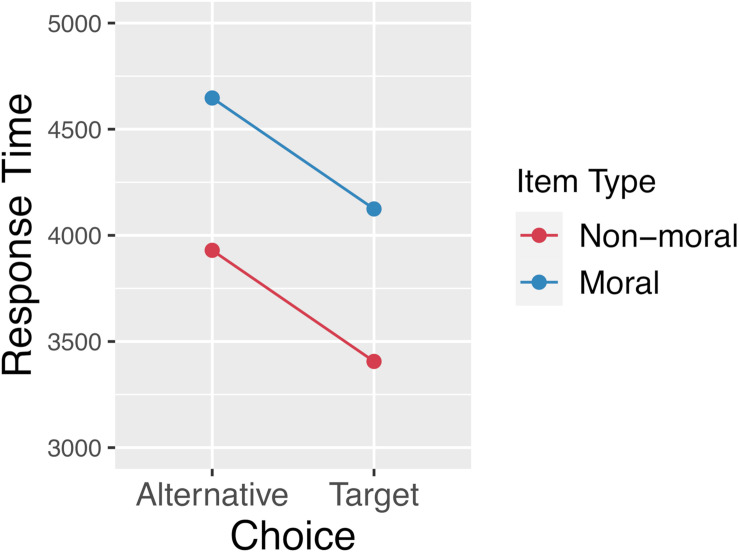
Response times by item type and whether the participant chose the target response option or the alternative. This plot reveals a main effect of item type, which is consistent with the fact that the moral stimuli were normed to be relatively equibiased with respect to initial preferences. There is also an effect of the velocity squashing manipulation, whereby movements toward the alternative are made slower than movements toward the target.

Our analysis excluded any trial with a response time greater than 2 standard deviations from the mean. 3.6% of trials were excluded on this basis. After exclusions, the mean overall response time was 4012 ms (SD = 2082 ms). The mean response time by item type and choice (target vs. alternative) is plotted in Figure 9. Response times were analyzed using linear mixed effects regression with the log-transformed response times as the dependent variable. A backward-fitting procedure was used to select the maximal random-effects structure justified by the data (Barr et al., 2013). The full model included item type (moral/normed vs. non-moral/un-normed), choice (whether the participant clicked the target or the alternative), and their interaction as fixed effects. Random-intercepts were included for participants and items, as well as by-subject random slopes for the effect of item-type. Model comparison revealed that moral items elicited slower response times than non-moral items [*b* = 0.2, *SE* = 0.04, *t* = 5.254, *X*^2^(1) = 23.54, *p* < 0.001], and that response times were slower when choosing the alternative relative to the target [*b* = −0.08, *SE* = 0.006, *t* = −14.533, *X*^2^(1) = 207.03, *p* < 0.001]. There was no interaction between item type and choice.

**FIGURE 10:**
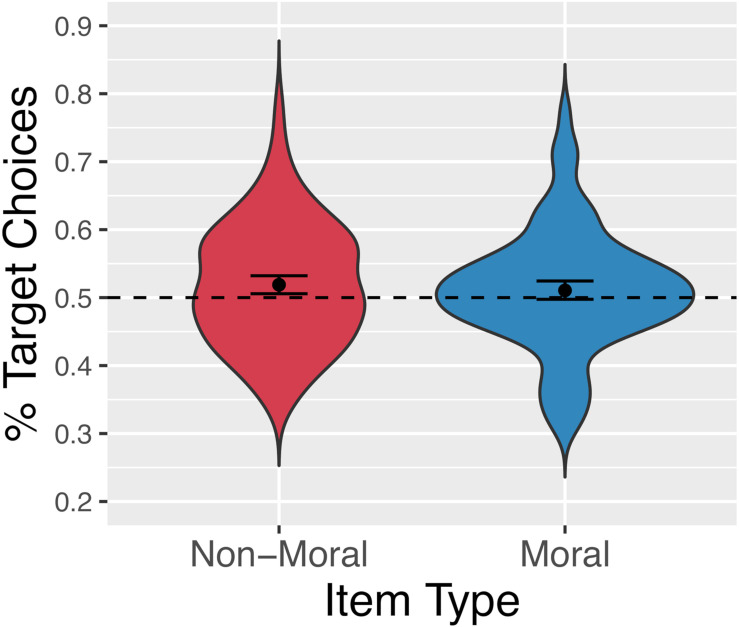
Percentage of trials on which participants selected the randomly pre-selected target option for non-moral (red, left) and moral (blue, right) statements.

Figure 10 shows the proportion of trials on which participants chose the target for each item type. Participants selected the target option 51.4% of the time overall, 51.06% of the time for our normed, moral items, and 51.9% of the time for our un-normed, non-moral items. The data were analyzed using logistic mixed-effects analysis. Again, a backward-fitting procedure was used to determine the maximal random-effects structure justified by the data. The full model included a single fixed effect, the intercept term. Random intercepts were included for participants and items, as well as random slopes for the effect of item type (normed/moral vs. unnormed/non-moral) by-participant. This analysis revealed a significant effect of adding the intercept term [α = 0.06, SE = 0.03, *z* = 2.03, *X*^2^(1) = 3.97, *p* < 0.05] and no significant effect of item-type, indicating that participants selected the target option more often than chance, and that this effect did not differ between moral items and filler items.

The overall mean rating for understanding was 6.52 (on a 1–7 scale; SD = 0.955), indicating that participants were able to read and understand both response options on most trials. For moral items, the mean understanding rating was 6.505 (SD = 0.92), for non-moral items it was 6.504 (SD = 0.99). Again using mixed effects linear regression, we analyzed the effects of item type, choice, and their interaction on confidence ratings. The full model included random intercepts for participants, and by-participant random slopes for the effect of item type. This analysis indicated no significant main effects or interactions.

The mean confidence rating was 5.28 (SD = 1.68) overall, 4.93 for non-moral items (SD = 1.81), and 5.64 for moral items (SD = 1.45). Using the same fixed and random-effects structure as noted above for the understanding ratings, we analyzed the effect of item type and choice on confidence. This analysis revealed a significant effect of item-type only, such that confidence was higher for moral items than for non-moral items [*b* = 0.644, *SE* = 0.046, *t* = 14.04, *X*^2^(1) = 100.8, *p* < 0.001].

To probe for detection of the mouse-cursor manipulation, the answers to the two post-experiment free-response questions were qualitatively explored. Several participants reported thinking that the mouse-cursor moved slower than normal in general – and indeed, the baseline cursor speed was already diminished below the default computer values (on a 2019 iMac Pro) to require participants to use overt arm movements rather than slight flicks of the wrist. However, no participants reported noticing a bias in the speed of movement toward one side or another.

### Discussion

In this experiment, we introduced a subtle, direction-specific bias in the velocity of the mouse cursor while participants decided which of two equidistant response options to click on. Although the effect is small, our results show that this manipulation was sufficient to bias decisions toward a randomly pre-determined target option, indicating that even moral decisions are sensitive to irrelevant influences on the motor system. Furthermore, no participants reported noticing this directional bias in cursor gain, so it is certainly possible that the strength of the manipulation could be increased further before becoming reliably detectable.

Response times also show the predicted effect of slower responses when choosing the non-target option, given the nature of the velocity squashing manipulation. The fact that response times were longer for the normed, moral stimuli is consistent with these stimuli being more equibiased than the un-normed, non-moral stimuli, and therefore requiring greater deliberation time. However, it should be noted that, in our experimental design, actual preferences cannot be assessed independently of the effect of the manipulation. This points to a potential weakness of our design, in that the statistical average of preferences in our population was used as a proxy for individual preferences. This leaves open the possibility that our normed stimuli were not actually less biased than the un-normed stimuli (although the results of Falandays and Spivey, 2020, and the main effect of item type on RTs are inconsistent with this possibility).

One notable finding was the presence of an effect of the manipulation on choices in *both* the moral stimuli, which had been normed such that response options themselves were relatively equibiased, and the non-moral stimuli, which had not been normed. Falandays and Spivey (2020), and our aDDM simulation above, suggest that – with Pärnamets et al. (2015b) gaze-contingent response-timing manipulation – only equibiased items (those where *A*≈ 0) may be influenced by the manipulation. However, our mDDM presented above straightforwardly accounts for the presence of an effect in both biased (those where *A* > > 0) and equibiased queries, in light of the motoric manipulation used here. While the gaze-contingent response-timing manipulation is only imposed when participants fixate both response options for a sufficient amount of time, which may not occur when response options are not equibiased, this motoric manipulation is imposed on all trials, leading to detectable effects even with a relatively weak velocity squashing factor and a relatively biased stimulus set. The more surprising finding that the normed stimuli did not show a *stronger* effect of the manipulation indicates either that our normed stimuli were not substantially less biased than our un-normed stimuli, or, more likely, that our experimental manipulation was rather subtle in practice, compared to the same velocity squashing factor in simulated space (see Figures 6, 7). This issue will need to be addressed by a follow-up study using a stronger velocity-squashing manipulation, with the predicted outcomes being a greater proportion of target choices and a greater difference in response times between target-choice trials and alternative-choice trials. It is also worth reiterating that our moral and non-moral stimuli differed in textual complexity, length, and other psycholinguistic dimensions. While this was a purposeful choice (Falandays and Spivey, 2020), future work will also need to account for the role of these differences in determining effect sizes in each stimulus condition.

## General Discussion

For decades, pollsters and psychologists have known that the way a question is worded can have a subtle but detectable influence on the response it induces (Schwarz, 1999). Once people make a choice (which is unavoidably biased by the way the question was delivered), their preferences tend to shift in favor of their chosen alternative (Sharot et al., 2010). In fact, even when those final choices are misrepresented in a sleight-of-hand, people will often fail to notice the misrepresentation and happily defend those choices as if they were actually their own (Pärnamets et al., 2015a; Strandberg et al., 2018).

Recent experiments that recorded eye movements during moral decision-making further show that a response can be manipulated by the timing and delivery of the response prompt. Inducing a response while the eyes are revealing a (potentially temporary) bias toward a particular option can have a subtle but detectable influence on choice (Pärnamets et al., 2015b; Ghaffari and Fiedler, 2018; Falandays and Spivey, 2020). In addition to that kind of *timing* perturbation, a *motor movement* perturbation can also influence choice, either through learning about movement costs (Hagura et al., 2017), or through “online” perturbations such as our velocity squashing manipulation. The present experiment and simulations suggest that the timing manipulation may depend significantly on the intrinsic preference among the choices being relatively equibiased, whereas a motor movement manipulation may be able to exert its subtle influence even on choices that start out far from equilibrium.

Although the effect on choice in our human experiment is small, the results of both the human and simulation experiments suggest that it is robust even with queries that are far from equilibrium. This finding shows that our decisions can be slightly influenced by even small biases present in the interface to a decision, even when those decisions deal with complex and personal issues like morality. If a bias of 1–2% above chance seems negligible at first, one need only consider the countless number of micro-decisions that most of us make each day with the help of a technological interface, and it quickly becomes apparent that these small nudges could add up to a massive difference over a relatively short period of time. The mouse cursor manipulation we used in our experiment was apparently undetected by *any* of our participants and is something that could be established on any website or phone application with nothing more than a few lines of code.

Furthermore, the points we are making should not be taken as applying strictly to decisions made using a mouse cursor or even a screen-based interface of any kind. Rather, it is the case that *every* decision occurs in the context of some constraints, whether it be a time pressure, a difference in the location of options or effort required to select them, or a bias in the accumulation of noise in preferences onto motor output. However, the degree to which these influences on our decisions can be *controlled* is certainly much greater in the case of human-machine interactions. As such, we hope these results will encourage our readers to understand the interface of a decision as being a potentially critical constituent of the decision itself, rather than a separate step that takes place after cognition has done its work.

Given the small size of this motoric influence on choice, if one was imagining that machine interfaces could be designed to substantially manipulate a specific decision by a specific person – for the common good or for selfish reasons – these results do not provide much support for that approach. Encouraging humans to support the common good, even when it means some degree of self-sacrifice, will still require training those humans to have good moral reasoning skills. There is no quick fix for that. Rather than interpreting these findings as evidence for a dystopian future where some particular high-stakes decision will be reliably manipulated by a smart phone that tracks a politician’s eye movements, there is a more realistic and scientific way to interpret these results.

At a theoretical level, it should be clear that these results simply could not happen so systematically if moral decisions were generated exclusively inside a neural module dedicated to moral reasoning – or even a network of such modules (e.g., Casebeer and Churchland, 2003). Instead, the evidence suggests that moral decisions (and potentially any difficult dilemma) emerge as a result of a human interfacing with their environment. While the majority of the statistical variance in those decisions is indeed determined by the human’s intrinsic preferences (Ghaffari and Fiedler, 2018), some portion of that variance is also determined by adventitious biases that take place in the interface itself. With human-machine interfaces becoming so ubiquitous, many of our everyday decisions – and some of our high-stakes decisions – are emergent results of this interaction between human and machine.

Our results can be situated within the vast literature on embodied cognition, which focuses on the important roles of the body, action, and motor systems of the brain in cognition more generally (Barsalou, 1999; Anderson, 2003; Clark, 2008; Shapiro, 2019). Work on decision-making in this framework has emphasized the role of “irrelevant” sensory information on judgments, such as the way that holding a heavier clipboard results in increased assessments of the importance of decisions (Jostmann et al., 2009), or the way that exposure to bad smells or disgusting rooms increases judgments of moral disgust with respect to crimes (Schnall et al., 2008; see also: Prinz, 2007).

Thus, as urged by Gibson (1979) and others, the domain of analysis when studying the human mind should not be solely the human organism itself but, instead, the entire organism-environment system. A human’s cognitive operations, their moral choices, their sense of self, perhaps even their consciousness, may be processes that are generated by the interaction of physical material both inside the skull and outside the skull (O’Regan and Noë, 2001; Clark, 2004; Aspell et al., 2009; Kirchhoff and Kiverstein, 2020; Spivey, 2020).

## Data Availability Statement

The datasets presented in this study can be found in online repositories. The names of the repository/repositories and accession number(s) can be found in https://osf.io/z9r47/ and https://osf.io/w26k3/.

## Ethics Statement

The studies involving human participants were reviewed and approved by the University of California, Merced IRB. The patients/participants provided their written informed consent to participate in this study.

## Author Contributions

JF and SS conducted the experiment. JF conducted the simulations. All authors contributed to the writing of the manuscript.

## Conflict of Interest

SS was employed by company Exponent. The remaining authors declare that the research was conducted in the absence of any commercial or financial relationships that could be construed as a potential conflict of interest.

## Footnotes

1 This simulation and replication were previously reported in a conference proceedings paper (Falandays and Spivey, 2020). The full stimulus selection procedure is reported below in the “Method” subsection of our current experiment, but the results of the prior human experiment are only described, for considerations of space.

2 https://osf.io/z9r47/

3 https://osf.io/w26k3/

## Supplementary Material

Supplementary Video 1 10 simulated trials plotted over time, using the same parameter settings as in Figure 3 (no preference). Timesteps have been approximately made equivalent to milliseconds.

Supplementary Video 2 10 simulated trials plotted over time, using the same parameter settings as in Figure 4 (maximum target preference). Timesteps have been approximately made equivalent to milliseconds.

Supplementary Video 3 10 simulated trials plotted over time, using the same parameter settings as in Figure 5 (maximum alternative preference). Timesteps have been approximately made equivalent to milliseconds.

## References

[B1] Abrams R. A. Balota D. A. (1991). Mental chronometry: beyond reaction time. *Psychol. Sci.* 2 153–157. 10.1111/j.1467-9280.1991.tb00123.x

[B2] Anderson M. L. (2003). Embodied cognition: a field guide. *Artif. Intell.* 149 91–130. 10.1016/s0004-3702(03)00054-7

[B3] Aspell J. E. Lenggenhager B. Blanke O. (2009). Keeping in touch with one’s self: multisensory mechanisms of self-consciousness. *PLoS One* 4:e6488. 10.1371/journal.pone.0006488 19654862 PMC2715165

[B4] Barr D. J. Levy R. Scheepers C. Tily H. J. (2013). Random effects structure for confirmatory hypothesis testing: keep it maximal. *J. Memory Lang.* 68 255–278. 10.1016/j.jml.2012.11.001 24403724 PMC3881361

[B5] Barsalou L. W. (1999). Perceptual symbol systems. *Behav. Brain Sci.* 22 577–660. 11301525 10.1017/s0140525x99002149

[B6] Barsalou L. W. (2016). Situated conceptualization offers a theoretical account of social priming. *Curr. Opin. Psychol.* 12 6–11. 10.1016/j.copsyc.2016.04.009

[B7] Beckerle P. Castellini C. Lenggenhager B. (2019). Robotic interfaces for cognitive psychology and embodiment research: a research roadmap. *Wiley Interdiscipl. Rev. Cogn. Sci.* 10:e1486. 10.1002/wcs.1486 30485732

[B8] Bogacz R. Brown E. Moehlis J. Holmes P. Cohen J. D. (2006). The physics of optimal decision making: a formal analysis of models of performance in two-alternative forced-choice tasks. *Psychol. Rev.* 113 700–765. 10.1037/0033-295x.113.4.700 17014301

[B9] Brainard D. H. (1997). The psychophysics toolbox. *Spatial Vis.* 10 433–436. 10.1163/156856897x00357 9176952

[B10] Casebeer W. D. Churchland P. S. (2003). The neural mechanisms of moral cognition: a multiple-aspect approach to moral judgment and decision-making. *Biol. Phil.* 18 169–194. 10.1023/a:1023380907603

[B11] Chemero A. (2011). *Radical Embodied Cognitive Science.* Cambridge, MA: MIT press.

[B12] Clark A. (2004). *Natural-born Cyborgs: Minds, Technologies, and the Future of Human Intelligence.* New York, NY: Oxford University Press.

[B13] Clark A. (2008). *Supersizing the Mind: Embodiment, Action, and Cognitive Extension.* New York, NY: Oxford University Press.

[B14] Clark A. Chalmers D. (1998). The extended mind. *Analysis* 58 7–19.

[B15] Ditzinger T. Haken H. (1995). “A synergetic model of multistability in perception,” in *Ambiguity in Mind and Nature*, (eds) Kruse P. Stadler M. Berlin: Springer, 255–274. 10.1007/978-3-642-78411-8_14

[B16] Dosher B. A. (1976). The retrieval of sentences from memory: a speed-accuracy study. *Cogn. Psychol.* 8 291–310. 10.1016/0010-0285(76)90009-8

[B17] Falandays J. B. Spivey M. J. (2020). “Biasing moral decisions using eye movements: replication and simulation,” in *Proceedings of the 42nd Annual Conference of the Cognitive Science Society*, (Austin, Tex: Cognitive Science Society), 2553–2558.

[B18] Fürstenau N. (2014). Simulating bistable perception with interrupted ambiguous stimulus using self-oscillator dynamics with percept choice bifurcation. *Cogn. Process.* 15 467–490. 10.1007/s10339-014-0630-4 25181991

[B19] Gallagher S. (2017). *Enactivist Interventions: Rethinking the Mind.* Oxford: Oxford University Press.

[B20] Ghaffari M. Fiedler S. (2018). The power of attention: using eye gaze to predict other-regarding and moral choices. *Psychol. Sci.* 29 1878–1889. 10.1177/0956797618799301 30295569

[B21] Gibson J. J. (1979). *The Ecological Approach to Visual Perception.* Boston, MA: Houghton-Mifflin.

[B22] Gidlöf K. Anikin A. Lingonblad M. Wallin A. (2017). Looking is buying. How visual attention and choice are affected by consumer preferences and properties of the supermarket shelf. *Appetite* 116 29–38. 10.1016/j.appet.2017.04.020 28433775

[B23] Hagura N. Haggard P. Diedrichsen J. (2017). Perceptual decisions are biased by the cost to act. *eLife* 6:e18422. 10.7554/eLife.18422 PMC5319835 28219479

[B24] Järvilehto T. (1998). The theory of the organism-environment system: I. description of the theory. *Int. Physiol. Behav. Sci.* 33 321–334. 10.1007/bf02688700 10333975

[B25] Jostmann N. B. Lakens D. Schubert T. W. (2009). Weight as an embodiment of importance. *Psychol. Sci.* 20 1169–1174. 10.1111/j.1467-9280.2009.02426.x 19686292

[B26] Kirchhoff M. D. Kiverstein J. (2020). Attuning to the world: the diachronic constitution of the extended conscious mind. *Front. Psychol.* 11:1966. 10.3389/fpsyg.2020.01966 32982832 PMC7475699

[B27] Kloosterman N. A. de Gee J. W. Werkle-Bergner M. Lindenberger U. Garrett D. D. Fahrenfort J. J. (2019). Humans strategically shift decision bias by flexibly adjusting sensory evidence accumulation. *eLife* 8:e37321. 10.7554/eLife.37321 PMC6365056 30724733

[B28] Kloosterman N. A. Kosciessa J. Q. Lindenberger U. Fahrenfort J. J. Garrett D. D. (2020). Boosts in brain signal variability track liberal shifts in decision bias. *eLife* 9:e54201. 10.7554/eLife.54201 PMC7398662 32744502

[B29] Krajbich I. (2019). Accounting for attention in sequential sampling models of decision making. *Curr. Opin. Psychol.* 29 6–11. 10.1016/j.copsyc.2018.10.008 30368108

[B30] Krajbich I. Armel C. Rangel A. (2010). Visual fixations and the computation and comparison of value in simple choice. *Nat. Neurosci.* 13 1292–1298. 10.1038/nn.2635 20835253

[B31] McElree B. Murphy G. L. Ochoa T. (2006). Time course of retrieving conceptual information: a speed-accuracy trade-off study. *Psychonomic Bull. Rev.* 13 848–853. 10.3758/bf03194008 17328384 PMC2323592

[B32] Neisser U. (1976). *Cognition and Reality: Principles and Implications of Cognitive Psychology.* New York, NY: W. H. Freeman publishing.

[B33] Newell B. R. Le Pelley M. E. (2018). Perceptual but not complex moral judgments can be biased by exploiting the dynamics of eye-gaze. *J. Exp. Psychol. General* 147 409–417. 10.1037/xge0000386 29355370

[B34] O’Regan J. K. Noë A. (2001). A sensorimotor account of vision and visual consciousness. *Behav. Brain Sci.* 24 939–1031. 10.1017/s0140525x01000115 12239892

[B35] Pärnamets P. Hall L. Johansson P. (2015a). “Memory distortions resulting from a choice blindness task,” in *Proceedings of the 37th Annual Conference of the Cognitive Science Society*, (Austin, Tex: Cognitive Science Society), 1823–1828.

[B36] Pärnamets P. Johansson P. Hall L. Balkenius C. Spivey M. J. Richardson D. C. (2015b). Biasing moral decisions by exploiting the dynamics of eye gaze. *Proc. Natl. Acad. Sci. U S A.* 112 4170–4175. 10.1073/pnas.1415250112 25775604 PMC4386374

[B37] Pärnamets P. Richardson D. Balkenius C. (2014). “Modelling moral choice as a diffusion process dependent on visual fixations,” in *Proceedings of the 36th Annual Conference of the Cognitive Science Society*, (Austin, Tex: Cognitive Science Society), 1132–1137.

[B38] Pezzulo G. Barsalou L. W. Cangelosi A. Fischer M. H. McRae K. Spivey M. (2013). Computational grounded cognition: a new alliance between grounded cognition and computational modeling. *Front. Psychol.* 3:612. 10.3389/fpsyg.2012.00612 23346065 PMC3551279

[B39] Prinz J. (2007). *The Emotional Construction of Morals.* New York, NY: Oxford University Press.

[B40] Putnam H. (1974). Meaning and reference. *J. Phil.* 70 699–711.

[B41] Ratcliff R. McKoon G. (2008). The diffusion decision model: theory and data for two-choice decision tasks. *Neural Comput.* 20 873–922. 10.1162/neco.2008.12-06-420 18085991 PMC2474742

[B42] Salthouse T. A. Ellis C. L. (1980). Determinants of eye-fixation duration. *Am. J. Psychol.* 93 207–234. 10.2307/1422228 7406068

[B43] Samaha M. Hawi N. S. (2016). Relationships among smartphone addiction, stress, academic performance, and satisfaction with life. *Comp. Hum. Behav.* 57 321–325. 10.1016/j.chb.2015.12.045

[B44] Schnall S. Haidt J. Clore G. L. Jordan A. H. (2008). Disgust as embodied moral judgment. *Personal. Soc. Psychol. Bull.* 34 1096–1109. 10.1177/0146167208317771 18505801 PMC2562923

[B45] Schwarz N. (1999). Self-reports: how the questions shape the answers. *Am. Psychol.* 54 93–105. 10.1037/0003-066x.54.2.93

[B46] Shadmehr R. Mussa-Ivaldi F. A. (1994). Adaptive representation of dynamics during learning of a motor task. *J. Neurosci.* 14 3208–3224. 10.1523/jneurosci.14-05-03208.1994 8182467 PMC6577492

[B47] Shadmehr R. Mussa-Ivaldi F. A. Bizzi E. (1993). Postural force fields of the human arm and their role in generating multijoint movements. *J. Neurosci.* 13 45–62. 10.1523/jneurosci.13-01-00045.1993 8423483 PMC6576309

[B48] Shapiro L. (2019). *Embodied Cognition.* Abingdon: Routledge.

[B49] Sharot T. Velasquez C. M. Dolan R. J. (2010). Do decisions shape preference? evidence from blind choice. *Psychol. Sci.* 21 1231–1235. 10.1177/0956797610379235 20679522 PMC3196841

[B50] Smith L. B. Thelen E. (2003). Development as a dynamic system. *Trends Cogn. Sci.* 7 343–348. 12907229 10.1016/s1364-6613(03)00156-6

[B51] Spivey M. (2007). *The Continuity of Mind.* New York, NY: Oxford University Press.

[B52] Spivey M. Fitneva S. Tabor W. Ajmani S. (2002). “The timecourse of information integration in sentence processing,” in *The Lexical Basis of Sentence Processing: Formal, Computational, and Experimental Issues*, eds Merlo P. Stevenson S. (Amsterdam: John Benjamins Publishing), 207–232. 10.1075/nlp.4.12spi

[B53] Spivey M. J. (2020). *Who You Are: The Science of Connectedness.* Cambridge, MA: MIT Press.

[B54] Spivey M. J. Grosjean M. Knoblich G. (2005). Continuous attraction toward phonological competitors. *Proc. Natl. Acad. Sci. U S A.* 102 10393–10398. 10.1073/pnas.0503903102 15985550 PMC1177386

[B55] Strandberg T. Sivén D. Hall L. Johansson P. Pärnamets P. (2018). False beliefs and confabulation can lead to lasting changes in political attitudes. *J. Exp. Psychol. General* 147:1382–1399. 10.1037/xge0000489 30148387

[B56] Tavares G. Perona P. Rangel A. (2017). The attentional drift diffusion model of simple perceptual decision-making. *Front. Neurosci.* 11:468. 10.3389/fnins.2017.00468 28894413 PMC5573732

[B57] Turvey M. T. Shaw R. E. (1999). Ecological foundations of cognition. I: symmetry and specificity of animal-environment systems. *J. Conscious. Stud.* 6 95–110.

[B58] Valliappan N. Dai N. Steinberg E. He J. Rogers K. Ramachandran V. (2020). Accelerating eye movement research via accurate and affordable smartphone eye tracking. *Nat. Commun.* 11:4553. 10.1038/s41467-020-18360-5 PMC7486382 32917902

[B59] Wojnowicz M. T. Ferguson M. J. Dale R. Spivey M. J. (2009). The self-organization of explicit attitudes. *Psychol. Sci.* 20 1428–1435. 10.1111/j.1467-9280.2009.02448.x 19818047

